# Study protocol for an online lifestyle modification education course for people living with multiple sclerosis: the multiple sclerosis online course (MSOC)

**DOI:** 10.1186/s12883-023-03298-0

**Published:** 2023-06-29

**Authors:** Jeanette C Reece, Maggie Yu, William Bevens, Steve Simpson-Yap, George Jelinek, Pia Jelinek, Rebekah Davenport, Nupur Nag, Kathleen Gray, Sandra Neate

**Affiliations:** 1 Neuroepidemiology Unit, Centre for Epidemiology and Biostatistics, Melbourne School of Population & Global Health, The University of Melbourne, Level 3, 207 Bouverie St Carlton, Melbourne, VIC 3053 Australia; 2 Menzies Institute for Medical Research, University of Tasmania, Hobart, Australia; 3 Melbourne School of Psychological Sciences, The University of Melbourne, Melbourne, Australia; 4 Centre for Digital Transformation of Health, The University of Melbourne, Melbourne, Australia

**Keywords:** Multiple sclerosis, Randomised controlled trial, Multiple sclerosis online course, Lifestyle, Behaviour change, Health-related quality of life

## Abstract

**Background:**

People living with multiple sclerosis (plwMS) seek access to information on evidence-based lifestyle-related risk factors associated with multiple sclerosis (MS). As the internet has made delivery of lifestyle information increasingly accessible and cost-effective, we designed the Multiple Sclerosis Online Course (MSOC) to deliver a multimodal lifestyle modification program for plwMS. Two MS online courses were developed: the intervention course based on lifestyle recommendations of the Overcoming Multiple Sclerosis (OMS) program and the standard-care course representing standard lifestyle recommendations from other MS websites. We examined for feasibility in a pilot randomised controlled trial (RCT), where satisfactory completion and accessibility were achieved across both study arms. From this success, a protocol for a larger RCT was developed to examine the effectiveness of MSOC in improving health-related quality of life (HRQoL) and other health outcomes in plwMS.

**Methods/design:**

This single-blinded RCT will recruit n = 1,054 plwMS. Participants in the intervention arm will receive access to a MSOC with seven modules providing evidence-based information on the OMS program. Participants in the control group will receive access to a MSOC of identical format, with seven modules providing general MS-related information and lifestyle recommendations sourced from popular MS websites, e.g. MS societies. Participants will complete questionnaires at baseline and at 6, 12, and 30 months after course completion. The primary endpoint is HRQoL, as measured by MSQOL-54 (both physical and mental health domains) at 12 months following course completion. Secondary outcomes are changes to depression, anxiety, fatigue, disability, and self-efficacy as measured by Hospital Anxiety and Depression Scale, Patient-Determined Disease Steps and University of Washington Self-Efficacy Scale, respectively, assessed at each timepoint. Further assessments will include quantitative post-course evaluation, adoption and maintenance of behaviour change from follow-up survey data, and qualitative analysis of participants’ outcomes and reasons for course completion or non-completion.

**Discussion:**

This RCT aims to determine whether an online intervention course delivering evidence-based lifestyle modification recommendations based on the Overcoming Multiple Sclerosis program to plwMS is more effective at improving HRQoL, and other health outcomes post-intervention, compared with an online standard-care course.

**Trial registration:**

This trial was registered prospectively with the Australian New Zealand Clinical Trials Registry, www.anzctr.org.au, identifier ACTRN12621001605886.

**Date of registration:**

25 November 2021.

## Background

Multiple sclerosis (MS) is an autoimmune degenerative disease of the central nervous system estimated to affect approximately 2.8 million people worldwide, with rising incidence [1]. Over 230 genetic variants [2] and multiple environmental factors, in particular Epstein-Barr virus [3], have been associated with MS onset, and multiple clinical and environmental risk factors have been associated with disease progression [2]. Several observational studies indicate modification of lifestyle-related risk factors, including smoking cessation, high diet quality, physical activity, adequate vitamin D level and sun exposure, and stress reduction, are associated with improved health outcomes, including improved quality of life [4–9] and disability and symptom burden [10–12], and reduced relapse rate [13, 14], depressive symptoms [10, 15], and fatigue [10, 16]. Therefore, a program presenting information on modification of these lifestyle risk factors may represent an effective, low cost and low risk intervention that could potentially improve outcomes for people living with MS (plwMS).

Previously, plwMS participating in an residential face-to-face lifestyle modification program based on the Overcoming MS (OMS) program [17], providing evidence-based recommendations regarding diet, exercise, stress management, sun exposure, and vitamin D [17], were found to have 11.3% and 19.5% improved overall quality of life at one and 5-year timepoints after workshop delivery, respectively [18]. Importantly, a study of a subgroup with more complete exposure and outcome data to the 3-year timepoint found that the participants demonstrated signifcantly improved health behaviours and reported clinically meaningful increases in quality of life at 1- and 3-year follow-up [19]. These findings suggest that participants at these face to face interventions implemented and sustained lifestyle changes and improvements in health outcomes were observed.

However face-to-face interventions are resource-intensive and present multiple potential barriers to participation, including financial, geographical, and disease-specific factors such as fatigue and mobility impairment [20]. The COVID-19 pandemic also highlighted that travel may not always be possible, and there are barriers to face-to-face learning for plwMS such as potential medication-associated immunosuppression and infection risk.

Existing online-based interventions have shown great efficacy at promoting positive lifestyle changes in a range of populations for a variety of medical conditions [21–26], including MS [27–30]. PlwMS are generally considered a highly motivated population who prioritise wellness and seek information on lifestyle [31], with favourable levels of engagement and retention demonstrated in learning programs both in the short- and long-term [32]. Therefore, alternative modes of delivery of education programs for plwMS such as an online resource could be both effective at delivering MS-related information and may overcome barriers of inaccessibility and cost. Currently there are few online resources available for plwMS regarding modification of lifestyle related risk factors. In addition, no MS-related online program has incorporated multiple risk factor modification recommendations into the one program, with previous online resources focused on interventions to increase MS-related knowledge and resilience [33] or single interventions such as physical activity [34, 35]. Moreover, none of the current MS-related online interventions have previously been tested for effectiveness in a large randomised controlled trial (RCT) with long-term follow-up.

As a result, our research team, together with a community advisory group of plwMS and an industry partner, developed the Multiple Sclerosis Online Course (MSOC) to test a comprehensive online program providing lifestyle-related risk factor modification information to plwMS, with the aim of improving quality of life and other health outcomes in plwMS. We developed two novel MSOCs that were designed to provide MS-related knowledge and teach skills and strategies for multiple lifestyle modifications. We used the established behavioural change theory of Social Cognitive Theory (SCT) based on four factors (self-efficacy, outcome expectations, goals and social-structural variables) [36], to assist participants to engage in health behaviour change [37]. The intervention course (IC) provides educational modules regarding the biological basis of MS and current best available evidence-based recommendations from the medical literature regarding diet, exercise, stress and stress-reducing activities, sunlight and vitamin D, omega 3, smoking cessation and alcohol intake based on the OMS program. The standard-care course (SCC) provides standard MS health recommendations from international MS websites. Both arms are identical in format and will be delivered in seven modules in an asynchronous manner via the same website. Information will be delivered multimodally via a mixture of written text, videos, and animations.

The MSOC IC and SCC were piloted for feasibility in a small RCT (the MSOC-Feasibility RCT) of 31 plwMS from Australia, New Zealand, USA, UK, Ireland and Canada (The University of Melbourne HREC 1851781.1). This study found comparable and satisfactory participant engagement in both the IC and SCC, with participants reporting course content to be readily accessible and enjoyable across both study arms [38–40]. Information obtained from quantitative and qualitative analyses of data from the MSOC-Feasibility RCT was then used to inform the redevelopment of course content and recruitment strategies, based on identified motivations for participation, to enable the MSOC to be tested for effectiveness in a larger RCT (the MSOC-Effectiveness RCT).

The MSOC-Effectiveness RCT aims to investigate the effect of the online education tool (MSOC) on health-related quality of life (HRQoL), and other health outcomes including depression, anxiety, fatigue, and disability in plwMS in the short- and long-term. Specifically, the MSOC-Effectiveness RCT will evaluate whether the MSOC IC results in improved HRQoL (the primary outcome) and other health outcomes (the secondary outcomes) compared with the MSOC SCC. Additionally, we will evaluate participants’ self-efficacy, engagement, adherence to lifestyle modification, course experience, and facilitators and barriers to course commencement and completion.

## Aims and hypotheses

We hypothesise that participants completing either arm of the MSOC will have improved physical and mental HRQoL due to the adoption of healthier lifestyle behaviours that address lifestyle related risk factors. However, participants in the RCT IC arm compared with the SCC arm will:

1. have clinically significant increased HRQoL at 6, 12 and 30 months, supporting previous reports of increased HRQoL following participation in a face-to-face lifestyle modification workshop based on the OMS program [19].
2. have clinically significant improvements in depression, anxiety, and fatigue at 6, 12 and 30 months. The cumulative effects of lifestyle modifications on reducing disability and relapses may not become evident until 30 months due to the time taken to reverse central inflammatory pathways [41].
3. adopt and adhere to some or all lifestyle recommendations in the short-term (6 and 12 months). In the SCC, there will be minimal changes in diet and physical exercise but there may be reduced smoking in current smokers.
4. demonstrate significant increases in self-efficacy as determined by quantitative analyses, as the course development was based on SCT [36]. Additionally, qualitative analyses may identify increased self-efficacy, control and confidence by IC participants, as these attributes have previously been identified as mediators of SCT and behaviour change [37].

The aims of the MSOC-Effectiveness RCT are to assess and compare the following between IC and SCC arms:

1. changes in HRQOL between baseline and the 6-, 12-, and 30-month follow-up (primary outcome).
2. changes in other health outcomes (depression, anxiety, fatigue, disability, and self-efficacy) between baseline and the 6-, 12-, and 30-month follow-up (secondary outcomes).

The secondary aims will be to:

3. assess changes in individual and collective lifestyle behaviours (defined as adherence to the recommended diet, omega-3 and vitamin D supplementation, moderate physical activity, sun exposure, and stress-reduction activities) between baseline and at the 6-, 12-, and 30-month follow-up.

Additional aims will be to:

4. assess changes in self-efficacy between baseline and at the 6-, 12-, and 30-month follow-up.
5. assess participant feedback of course by post-course evaluation surveys.
6. explore participants’ self-efficacy, control and confidence, as well as the facilitators and barriers to course completion through qualitative interviewing of a subset of participants across both study arms.

## Methods/design

The present study is a CONSORT-R compliant RCT that is registered with the Australian New Zealand Clinical Registry (ACTRN12621001605886).

The trial (named the MSOC-Effectiveness RCT) will be carried out as a single-blinded RCT study design comparing the MSOC IC and SCC. The 33-item Standard Protocol Items: Recommendations for Interventional Trials (SPIRIT) guidelines were followed in the development of the study protocol [43].

Written informed consent will be obtained from all participants.

This study was approved by The University of Melbourne Human Research Ethics subcommittee (ID: 22,140).

All methods were carried out in accordance with relevant guidelines and regulations.

### Participants

Participants will comprise people over the age of 18 years with a self-reported medically confirmed diagnosis of MS. Participants will be recruited online and the MSOC and questionnaires will be delivered online. Although participants will be internationally recruited, this is a single-site study as the study will be administered and conducted solely at the Neuroepidemiology Unit at the Centre for Epidemiology and Biostatistics, Melbourne School of Population and Global Health, The University of Melbourne. The single-site nature of the study guarantees that all participant data will be securely stored in one location, The University of Melbourne server.

### Patient and public involvement, and partnerships

To ensure representation of the MS community during course design and development, a community advisory group was assembled by invitation through the Australian Facebook group ‘People Living with MS’. This group of volunteers, with representatives across MS types and disease duration, met face-to-face and online with one researcher (WB) throughout the study design and development phase. They discussed and assisted with decision-making regarding key features of the MSOC including design, content, and delivery. The MSOC was then developed in partnership with this community advisory group, the research team, and a web design and development partner.

### Participant recruitment

Participant recruitment will be via online invitations published on MS society websites throughout Australia, New Zealand, USA, UK, Ireland, and Canada, and via Facebook support groups (e.g., MS Peer Support Group), research newsletters (e.g., Clinical Trials Australia), and Instagram. Study news and information were posted on a study Facebook page available to the public (https://www.facebook/MSOCresearch). Interested participants will provide their name, email address, and answer two questions; (1) I am over 18 years of age (yes or no) and (2) I have a neurologist’s diagnosis of Multiple Sclerosis (yes or no).

Participants will be screened for eligibility on the registration website.

#### Inclusion criteria

1. Be 18 years old or over;
2. Have a neurologist-confirmed diagnosis of MS.

#### Exclusion criteria

Participants fulfilling the following criteria will be able to complete the course but will not be utilised in analyses.

1. Experiencing any serious comorbid chronic illness or neurological illness/injury other than MS that would threaten regular participation or significantly affect the outcome measures in its own right, such as motor neurone disease or stroke, as determined by the study investigators;
2. Currently participating in another RCT.

#### Participant information sheet

Information regarding the nature of the RCT was provided in a participant information sheet provided to plwMS interested in participating in the RCT. This information outlines that two courses have been developed (an IC and SCC), and the RCT aims to test the effectiveness of the IC, and that participants will be randomised to one of the two courses and remain blinded to their allocation.

#### Randomisation

Study advertisements will contain a link to the study landing webpage (https://www.msonlinecourse.com.au/). There, the registration form requires the name and email address and asks questions assessing inclusion criteria. Those meeting the inclusion criteria will be registered as ‘pending’ participants and emailed a link to set up a password to the course. Registered participants will be assigned to either the IC or SCC group at a ratio of 1:1 using simple randomisation. The randomisation sequence is computer-generated and implemented through the course website (https://app.msonlinecourse.com.au/).

#### Baseline assessment and consent

Upon account setup, participants can access the ‘Welcome to the MSOC study’ module in the course platform (https://app.msonlinecourse.com.au/login). The Welcome to the MSOC study module contains the Plain Language Statement and a link to the baseline survey (https://melbourneuni.au1.qualtrics.com/jfe/form/SV_cHNCN0bwKWn3cq2) which participants are encouraged to complete before the course starts. Participants are asked to affirm their consent to participate in the research study on the first page of the baseline survey. The baseline survey comprises 166 questions and has been estimated to take 45–60 min to complete. This survey queries participant demographics, health behaviours, and clinical outcome measures, and will form the baseline data which subsequent data will be compared with. Participants can commence Module 1 (‘Welcome to the MSOC study’) after completing the baseline survey (Table 1).

**Table 1:** Summary of recommendations of the Intervention Course (IC) arms and Standard Care Course (SCC) arms

Week	Modules	IC content and recommendations	SCC content and recommendations
1	1. Introduction to the course	Welcome to the course, including how to proceed, and navigate what to expect, and to outline the endpoints for participants. An overview of MS will also be provided.	Welcome to the course, including how to proceed, how to navigate, what to expect, and to outline the endpoints for participants. An overview of MS will also be provided.
	2. Eat well	Evidence behind the role of diet in MS risk, disease activity, and QOL, including saturated fat and its relationships with MS onset and progression. Recommends a plant-based wholefood diet plus seafood, with < 20 g/day saturated fat, as well as omega-3 fatty acid supplement use, or 20–40 ml of flaxseed oil (or equivalent) per day. Evidence supporting moderate caffeine and alcohol intake in MS	Information regarding the importance of a balanced diet. Public Health England Eatwell guide presented. Information from the US National MS society presenting a selection of diets used by people with MS including gluten free, Palaeolithic diet, McDougal diet, Mediterranean diet and Swank diet. Alcohol consumption should follow national guidelines.
2	3. Sunlight and vitamin D	Detailed information about how vitamin D is made from skin exposure to sunlight, along with evidence supporting the potential role of vitamin D in MS risk and progression Recommendations for optimal levels of vitamin D supplementation and blood levels are presented. Recommends sun exposure of at least 15 min per day, 3–5 times a week, and vitamin D3 supplement use of at least 5,000 IU per day.	Information regarding Vitamin D and MS development, latitude gradient of MS and how the body produce vitamin D from sunlight. No specific recommendations on sun exposure or supplementation described. Three options presented: wait until more information is available, supplement ‘blindly’ or supplement if blood 25-hydroxyvitamin D levels are low.
	4. Exercise	The benefits of exercise (neurological, cognitive, physical), how and why to implement an exercise plan with video examples provided. Recommends 20–30 min, 5 times/week exercise outdoor preferably.	Presented the critical role exercise plays in MS. Recommend 30 min or more of moderate aerobic activity and strength training at least twice per week
3	5. Meditation and use the mind-body connection	Introduction to mental health and relevance in MS, to the science behind stress and its link to inflammation/MS. Mind-body connection, meditation and other stress reduction techniques along with a guided meditation video are delivered along with how to develop a mental health and wellbeing improvement strategy. Recommends 30 min or more of daily meditation.	No conclusive link between stress and MS, as the evidence to date is contradictory. Introduction to the associations between MS and chronic distress and stressful life events.
	6. Medication and family prevention	Genetic, smoking, dietary and vitamin D related risks in MS development/progression. MS and pregnancy and breast feeding. Role of medication in MS discussed. Recommends minimize risks to self, family and during pregnancy via diet and vitamin D, no tobacco smoking and avoid passive smoke exposure	Genetic risk of getting MS. Smoking increases the risk of MS. The role of medication in MS discussed.
4	7. Review and consolidation	Program overview and recap, and an outline of next steps to take. Follow-up questionnaires for longitudinal follow-up discussed and participants asked to complete, and link to forums for further engagement (aimed at enhancing retention). Concluding remarks and closing ceremony.	Program overview and recap. Follow-up questionnaires for longitudinal follow-up discussed and participants asked to complete, and link to forums for further engagement (aimed at enhancing retention).
5/6	Catch-up	Participants have 2 weeks to complete any modules missed.	Participants have 2 weeks to complete any modules missed.

### Study arms

#### Intervention vs. standard care course

The IC and SCC were each designed and developed by the research team. The study arms differed only in content as shown in a summary of course material presented in the separate study arms (Table 1).

Both courses are delivered in 7-module, self-administered programs in an asynchronous manner over a 6-week period. Modules are released as per a pre-defined schedule, 2 modules per week for 3 weeks and one module in week 4, with 2 weeks added for completion. Future modules are gated until completion of preceding modules, but completed modules are available for review throughout the entire 6-week period.

#### Intervention course content

The IC content was adapted from the evidence-based lifestyle modification program, the OMS program [17]. Content across the seven modules includes an introduction to MS pathophysiology followed by the lifestyle recommendations: diet, exercise, stress and stress-reducing activities, sunlight and vitamin D, omega 3, smoking cessation, moderate alcohol intake. Additionally, there is a module with information to assist in preventing MS in family members. (Table 1)

#### Standard care course content

The content of the SCC was sourced entirely from MS Society websites in the public domain, including Multiple Sclerosis Australia, Multiple Sclerosis Research Australia, National MS Society, Multiple Sclerosis Society UK, Multiple Sclerosis Society of Canada with text, video, and image/animation content compiled into modules to mirror the IC modules. The SCC aims to reproduce the advice that plwMS typically receive during medical consultations and online advice from MS Societies (Table 1).

In both the IC and SCC, participants can create their own profiles with photos and any personal information they wish to share for purposes of building of an online community. Community insights deliver results of polls of participants motivations to undertake the MSOC and where they heard about the study from within modules for interest only. A community forum is moderated by one researcher in each arm where participants are encouraged to engage with the moderator and other participants, ask questions, and share experiences. A resources tab enables the sharing of interesting resources.

### Outcomes and assessments

The number of questions asked of participants and the timing of these queries is described in Table 2.

**Table 2:** Number and timing of querying of demographics, exposures and outcomes

			Timeframe after completion of MSOC					
Assessment/Measure	No. Questions	Screening	Baseline	Completion	One month	6 months	12 months	30 months
Informed consent	1		X					
***Demographics***								
Age	1	X	X					
MS type diagnosed	2	X	X			X	X	X
MS duration	2	X	X			X	X	X
Do you follow a MS-specific lifestyle program?	2	X	X			X	X	X
Do you follow a MS-specific diet	2	X	X			X	X	X
Sex and gender	2		X					
Residential address and country	1		X					X
Country of birth	1		X					
Height/weight	2		X			X	X	X
Comorbidities (yes and specify)	2		X			X	X	X
Marital status	1		X			X	X	X
Education	1		X					
Employment status	1		X			X	X	X
Alcohol and smoking	5		X			X	X	X
Medications	4		X			X	X	X
Perceived Social Support	12		X			X	X	X
***Exposures***								
Physical activity: IPAQ-SF	7		X			X	X	X
Meditation: MAQ	3		X			X	X	X
Sun exposure	4		X			X	X	X
Diet quality: DHQ	22		X			X	X	X
Consumption of meat or dairy	2		X			X	X	X
Omega-3 intake: dose, frequency	2		X			X	X	X
Vitamin D intake: dose, frequency	2		X			X	X	X
***Health outcomes***								
HRQOL: MSQOL-54	54		X			X	X	X
Disability: PDDS	1		X			X	X	X
Anxiety and depression: HADS	14		X			X	X	X
Fatigue: FSS	9		X			X	X	X
Self-efficacy: UWSE-6	6		X			X	X	X
Qualitative interviewing	(7)				X		X	
If clinically significant improvement in HRQOL subset scores in Intervention group from baseline, we will consider an amendment to the RCT to provide participants in the SCC access to the IC							X	
If no clinically significant improvement in HRQOL subset scores in IC from baseline, the RCT will not be amended							X	
Total no. quantitative data questions			166			154	162	158
(not including qualitative)								

### Quantitative assessments of exposures and outcomes

Participants will complete a baseline questionnaire at the commencement of the course. The same questionnaire will be administered 6-, 12-, and 30-months after course completion.

#### Demographics

##### Demographic data

date of birth, sex, self-defined gender, current location of residence, country of birth, cultural background (Australian Standard Classification of Cultural and Ethnic Groups [44]), highest education level, marital status, and employment status will be queried.

#### Exposures

##### Body mass index (BMI; weight/height^2^)

calculated from self-reported height (cm or in) and weight (kg or lb.) and categorised according to World Health Organisation cut-offs [45].

##### Diet quality

measured using a modified DHQ [46], with three questions related to salt and alcohol intake and one on alcohol omitted, as described previously [4, 8, 47]. For questions related to meat consumption and the trimming of fat from meat and the frequency of consuming processed meat, an option of “I do not consume meat” was added. An option of “I do not consume dairy” for the question related to low-fat dairy intake was also added. These questions enable dichotomous terms for meat and dairy consumption to be generated. Responses for each DHQ question are scored and summated and the total DHQ score expressed as a scores of 100. In addition to the total DHQ score, 8 subscores are calculated and expressed as scores out of 100: Cereal, Fruit/Vegetable, Takeaway foods, Fat, Omega-3, Fibre, Food Choices, and Food Preparation.

##### Physical activity

measured using the 7-item International Physical Activity Questionnaire (IPAQ) used in other MS studies [48], to estimate the frequency and duration of vigorous and moderate intensity activities, as well as walking. Total physical activity is measured by weighting each type of activity according to respective energy requirements defined by METs (multiples of the resting metabolic rate). MET estimates of IPAQ are categorised as Inactive, Minimally Active, and Active according to IPAQ guidelines [48].

##### Omega-3 and vitamin D supplementation

type and daily dosage of omega-3 supplementation used on average in the last 6 months will be queried. Types of omega-3 supplementation include fish oil, high potency fish oil, and plant-based omega-3 sources such as flaxseed oil. Vitamin D supplementation is queried by dosage and frequency of vitamin D supplement intake.

##### Sun exposure

number of days per week and average duration of time (none, 1–15 min to > 60 min) spent in summer and winter. Participants will also be asked whether they deliberately increased their sun exposure with the goal of increasing their vitamin D levels.

##### Tobacco smoking and alcohol

assessed by querying self-reported tobacco smoking status (never/ex-smoker/current smoker) by querying age of commencing smoking, numbers of cigarettes smoked per day (< 1 to > 20), and for ex-smokers, age of smoking cessation (< 6 months to 10 years or longer). Alcohol consumption frequency (15 categories from never drank alcohol to everyday) and quantity (11 categories of average number of standard drinks from 0 to 10 + when drinking alcohol) will be queried and then categorised as none/limited/heavy according to World Health Organisation cut-offs [49].

##### Stress management

two questions of the Mindfulness Adherence Questionnaire (MAQ) [50], a 6-item subset of the main questionnaire, will be used to measure the type and frequency of meditation.

#### Health outcomes

##### HRQoL

HRQoL will be measured by MSQOL-54 which has been psychometrically validated in plwMS and will be used to assess a spectrum of HRQOL outcomes. The two primary outcome measures will be change in Physical Health Composite (PHC) and Mental Health Composite (MHC), estimated from MSQOL-54 scores of relevant subscales as per MSQOL-54 guidelines [51]. Although minimal clinically important differences have not been established for the MSQOL-54 composite scores, differences of at least five points have previously been determined as the minimum clinically meaningful change in a HRQOL measure [52, 53]. This is based on recommendations that a difference equivalent to half the standard deviation be universally considered an important magnitude for all HRQOL tools [54].

##### Fatigue

Clinically significant fatigue will be assessed by the 9-item Fatigue Severity Scale (FSS) [55]. The FSS has good internal consistency, stability, and sensitivity to change over time [56, 57]. A mean score > 5 is widely used for plwMS to indicate clinically significant fatigue [57–59]. A meaningful change in the FSS has been reported as a change of ≥ 1.9 points in plwMS [60] and will be considered clinically meaningful here.

##### Anxiety and depression

The Hospital Anxiety and Depression Scale (HADS) [61] will be used to assess the presence and severity of anxiety and depression symptoms and provides scores for anxiety and depression, with a 2-point change in subscores indicating a clinically relevant change [62].

##### Patient-reported disability

Disability will be assessed using the Patient-Determined Disease Steps (PDDS) [63], a self-reported measure of ambulatory disability that correlates well with the Expanded Disability Status Scale [64]. The PDDS is considered a practical tool to assess changes in disability over time and scored ordinally from 0 (normal) to 8 (bed bound) with detailed descriptors [65]. One step will be considered a clinically meaningful change in the PDDS. PDDS will also be used to estimate the disease duration-adjusted Patient-determined MS Severity Score (P-MSSS) [66] that will be evaluated as both a continuous and categorised variable [normal/mild (P-MSSS ≤ 3), moderate (P-MSSS > 3–6), and severe (P-MSSS > 6)].

##### Self-efficacy

Self-efficacy, the perception that behaviour change could result in improved outcomes and personal recognition that people have the ability to bring about behaviour change [37], has been recognised as an important component of behaviour change. Self-efficacy will be measured using the University of Washington Self-Efficacy (UWSE) survey, a psychometrically sound instrument that includes 6 items and which has been validated in plwMS [67]. Self-efficacy using the UWSE-6 will be assessed as a continuous term and dichotomised at the median as there is no established cut off point indicating sufficient self-efficacy.

#### Other clinical characteristics

MS type will be queried as benign/relapsing-remitting/secondary-progressive/primary-progresive/progressive-relapsing or unsure. Date of MS diagnosis and disease onset will be queried and used to calculate disease duration from MS diagnosis and onset, respectively. At baseline, number of treated comorbidities will be queried using the Self-administered Comorbidity Questionnaire [68].

#### Medications

Medications used will be queried, including currently used disease modulating therapies (DMTs) immunomodulatory medications for MS (alemtuzumab, cladribine, daclizumab, dimethyl fumarate, fingolimod, glatiramer acetate, interferon-beta, laquinimod, natalizumab, rituximab, teriflunomide, ocrelizumab, ofatumumab and other DMT), as well as prescription antidepressant, medications used for fatigue, and anxiolytic/sedative medications.

### Quantitative assessment of course completion and satisfaction

A 5-minute evaluation survey will be emailed to registered participants at the completion of the course to rate their experience with the study. Participants are first asked if they have (1) started and (2) (if started) completed the course. Three series of questions are then asked depending on their answers: non-starters, non-completers, and completers.

#### Non-starters: barriers to commencement

Participants who did not start the course are asked:

- Barriers or issues that hindered you from starting the course [e.g., technical issues, difficulty with the enrolment process, MS-related health issues, or/and other (specify)]

#### Non-completers: barriers, satisfaction, and outcomes

Participants who started the course but did not complete the modules are asked:

- Barriers or issues that hindered you from completing the course [e.g., technical issues, irrelevant course content, content not pitched at a suitable level, course presentation, health, time, family, or work issues, or/and other (specify)]
- Course evaluation:
  - The overall experience of the course.
  - How likely are you to recommend the course to a family/friend with MS?
  - Topics about which you would have liked some or additional information.
  - Participation in and usefulness of forums.
- How familiar were you with the content of each module prior to the course?
- How likely are you to change each lifestyle behaviour in the course modules (e.g., diet, exercise) because of taking the course?

#### Completers: motivation, satisfaction, and outcomes

Questions for participants who completed the course include:

- Where did you first hear about the study (e.g., MS society websites).
- Motivations for completing the course (e.g., to gain information MS and/or lifestyle behaviour, opportunity to participate in MS research, etc.)
- Course evaluation:
  - The overall experience of the course.
  - How likely are you to recommend the course to a family/friend with MS?
  - Topics about which you would have liked some or additional information.
  - Participation and usefulness of forums.
- How familiar were you with the content prior to the course?
- How likely are you to change lifestyle behaviours (e.g., diet, exercise) because of completing the course?

### Qualitative assessments

Two qualitative studies, using semi-structured interviews, will be conducted as part of the broader project, which aim to explore participants’ self-efficacy, control and confidence, as well as the facilitators and barriers to course completion through qualitative interviewing of a subset of participants across both study arms.

#### Course completers: motivation and outcomes

The first qualitative study is an investigation of the motivations and outcomes experienced by participants who completed the course. Eligibility includes completion of the MSOC and the baseline and post-course evaluation surveys. Interviews will be undertaken 4–8 weeks and then 12 months after completing the course. Eligible participants from the IC and SCC arms will receive an email requesting their participation in a 30–60 min semi-structured interview until there are approximately 25 interviewees from each arm. Interviews will be conducted online by four trained interviewers.

The 4–8-week interview will cover the following domains:

- Participants’ motivations to undertake the course.
- Views about the content of the course.
- Description of modification of lifestyle behaviors undertaken since course completion.
- Changes in self-efficacy including changes to motivation, confidence, control, and attitudes to MS.
- Changes in perceptions of wellbeing, and physical and mental health.
- Engagement with the community forum.

The 12-month interview will cover the following domains:

- Adoption and maintenance of lifestyle recommendations of respective courses.
- Changes in self-efficacy including changes to motivation, confidence, control and attitudes to MS.
- Changes in perceptions of wellbeing, and physical and mental health.

#### Course non-completers: reasons for non-completion

The second qualitative study will explore reasons for non-completion of the MSOC at different timepoints. Interviews will be undertaken at the completion of the 6-week course (to give all participants the opportunity to start and complete the course within the 6-week period that the course is running).

For this analysis, purposive sampling will be used to engage with participants in four distinct categories from participants in the IC and SCC arms:

- Signed up but did not create a profile.
- Created a profile but did not commence the course.
- Created a profile but only completed module 1.
- Created a profile but only completed module 2.

These interviews will cover the domains:

- Reasons for non-completion.
- Motivations and expectations of the course.
- Digital health experience and literacy.
- How people gather information about MS.
- Strategies/MSOC amendments how the course could be improved to increase completion rates.

**Fig. 1:**
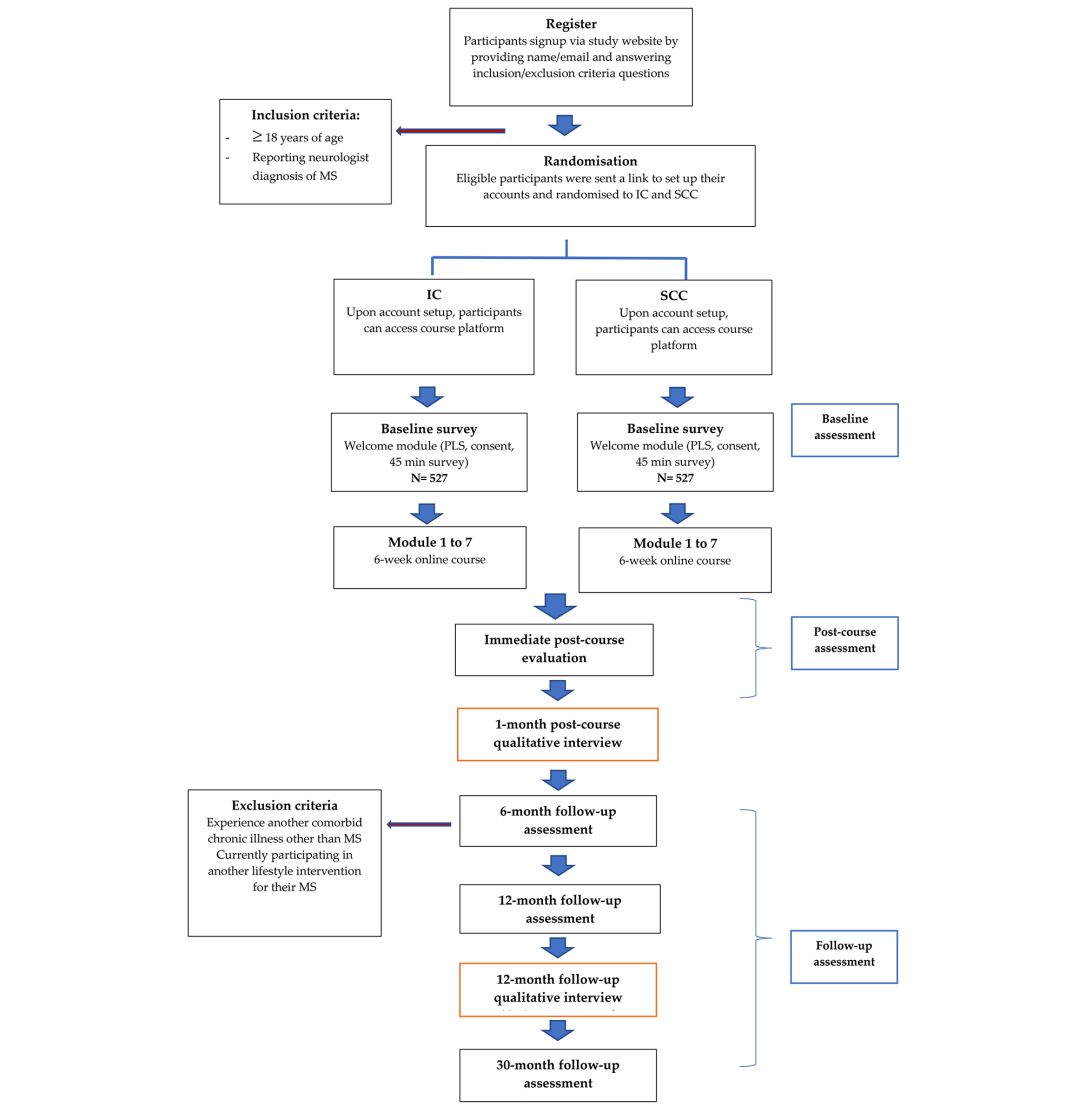
Flow-chart of RCT MS - multiple sclerosis; IC - intervention course; SCC - standard care course; PLS - plain language statement

The flow chart of the conduct of the RCT and follow up assessments is shown in Fig. 1.

### Data collection, management, and analysis

#### Quantitative data

##### Analytic data

will be collected via the backend of the course platform and Google Analytics. Excel files of administrative data such as number of participant logins and course progress will be downloaded from the course platform (https://app.msonlinecourse.com.au/admin/). Google Analytics also provides data tracking on how participants interact with the online course, such as average time spent per page, what interactive elements participants engaged with, and what pages participants commonly exited the course on, among others. The analytics integrated into the platform itself will allow investigators to analyse the number of participants that drop out over the length of the course and at what points they do so.

##### Exposures and health outcome quantitative data

will be collected via validated questionnaires completed by participants at specified timepoints (Fig. 1; Table 1). Participants who experienced another comorbid chronic illness other than MS or participated in another lifestyle intervention will be excluded from analysis.

#### Sample size

Numbers of participants necessary for sufficient statistical power (80%) were calculated to detect a 5-point difference in the PHC and MHC between the IC and SCC. To further account for loss-to-follow-up across the study period, based upon results from the MSOC-Feasibility RCT, we anticipate a 42% loss-to-follow-up between randomisation and 12-month follow-up. It is likely this is an overestimate of what will be observed in the proposed MSOC–Effectiveness RCT due to redevelopment of the MSOC based upon feasibility data.

Taking these factors into account, we estimate 1,054 participants, 527 in each arm (1:1 allocation) with data at baseline and follow-up would give 80% power to detect a 5-point change in HRQOL across both PHC and MHC, allowing for 42% attrition. This estimation of power was undertaken with Bonferroni correction and is based on the mean (and SD) of PHC and MHC scores between groups at baseline.

#### Statistical methods

Linear regression will be used to determine cross-sectional and prospective relationships with HRQOL. Characteristics of disability will be assessed using linear regression. Characteristics of dichotomous clinically significant fatigue, depression, and anxiety, will be assessed by log-binomial regression. Characteristics of self-efficacy composite scores will be assessed by linear and log-binomial regression for continuous and dichotomised terms, respectively. All models will be assessed for potential confounders and appropriate adjustments will be made in analyses.

### Qualitative data

Participants will be invited by email to participate in a 30-45-minute researcher-developed qualitative interview, commencing four weeks after course completion. Interviews will be conducted via telephone or video conference. Audio recordings of interviews will be de-identified and transcribed for analysis via voice recognition software (www.temi.com), edited by researchers and stored securely on The University of Melbourne server. Nvivo Software (www.qsrinternational.com) will be used to facilitate data management. Data analysis will be conducted within a qualitative paradigm using reflexive thematic analysis [69]. Reflexive thematic analysis is considered the most appropriate analytic process due its lack of grounding in a particular philosophy [70], and as a method suitable to exploring people’s experiences, views, and perceptions, and allowing expression of results in a way accessible to those in the wider community [71]. Reflexive thematic analysis also emphasises the importance of the researcher being deeply involved in the research. Data will be analysed by the process of data familiarisation, coding, and theme development and refining. Data extracts (quotes) will illustrate themes.

### Trial status

The RCT was registered at Australian New Zealand Clinical Trials Registry (ACTRN12621001605886) on 25 November 2021. Protocol version 4.0, dated 16 March 2023. Recruitment commenced on 23 June 2022. As of June 2023, four rounds of recruitment for participants in the MSOC trial have been completed, and further rounds will be scheduled until the estimated sample size is fulfilled. Recruitment for qualitative evaluation will occur in June and December 2023.

## Discussion

The MSOC was developed to translate an intensive face-to-face residential workshop delivering lifestyle modifications based on the OMS program for plwMS into an online format to overcome resource and accessibility barriers. We outline the protocol of a large international RCT aimed to examine the effectiveness of the MSOC in improving HRQoL (the primary outcome) and other health outcomes (the secondary outcomes) in the IC compared with the SCC. The MSOC is the only online education resource providing information on comprehensive lifestyle-related risk factor modification available and this RCT will be the first to examine the long-term effects of the online intervention on HRQoL and other health outcomes in quantitative analyses. Additionally, qualitative analyses will explore participants’ self-efficacy, control and confidence, as well as the facilitators and barriers to course completion.

Before commencing online course development, a systematic review and meta-analysis of RCTs (n = 32) describing digital health self-management interventions for plwMS was conducted to inform likely participant attrition. The pooled attrition rates for the intervention and control arms were 14.7% and 15.6%, respectively [72], demonstrating no significant difference in attrition between groups. A second systematic review and meta-analysis of RCTs of lifestyle interventions in other chronic diseases found no evidence of differential attrition between intervention and control arms, increasing confidence in conducting such studies with minimal potential of attrition bias [73].

After development of the MSOC, the pilot feasibility study (MSOC-Feasibility RCT) was conducted to quantitatively assess the feasibility of the MSOC using Likert scales [39] and found the MSOC performed well in accessibility, learnability, and desirability domains. Analysis of the primary outcome of feasibility (whether plwMS completed the MSOC) found 59% completion rates in the IC arm vs. 50% in the SCC arm, which confirmed course feasibility. Qualitative analysis of participant interviews indicated that several had implemented some lifestyle behaviour recommendations, however differences between the IC and SCC were not examined [38, 40]. Collectively, these results were used to inform the course redevelopment and recruitment strategies.

The present RCT was designed to assess differences in HRQoL and health outcomes between participants in the IC compared with the SCC. However, design of this RCT presented challenges. As information provided in the IC is freely available on the internet, there is the possibility of contamination of information to those in the SCC. A further issue may arise, because in some peoples’ eyes, participants in the SCC may be denied access to the best standard of care available or, in this case, the most up-to-date and complete knowledge base. However, we argue that the information provided in the IC has not been previously proven in an RCT to be more effective at improving health outcomes than the information in the SCC. Hence, we will conduct the present RCT to address this question. Further, as it is generally considered that if a state of ‘clinical equipoise’ exists, where there is a state of ‘honest professional disagreement’ among healthcare professionals about which treatment (in this case, knowledge) is best [74], then randomisation to the SCC arm will constitute a ‘fair bet’ that both arms are *a priori* equally valuable [75]. As such, the concept of clinical equipoise was adopted by the researchers to ethically rationalise the randomisation process. All SCC participants will be provided with a link to the IC at trial completion to ensure that all participants have access to the IC.

Moreover, consent to participate in the RCT will be voluntary and participants will be adequately informed [76]. While the concept of adequate information is difficult to define, and should generally include the understanding of the risks and benefits of the IC and also the concept of blinding and randomisation [76], participants will be provided with all the information practically possible in the participant information sheet for informed consent, while also balancing the risk of patient unblinding, as supported by the approval of the Human Research Ethics Committee [75].

HRQoL was chosen as a primary endpoint for several reasons. We acknowledge that HRQoL may be influenced by factors not related to lifestyle changes. However, HRQoL has been used extensively as a patient-reported outcome measure in MS-related observational studies [8, 18, 19, 77, 78] and is a well-established measure in MS-related clinical trials [79]. Further, observational studies of heterogeneous MS cohorts found lifestyle risk factors were robustly associated with HRQoL, independent of other factors such as disability, fatigue, and relapse rate [8, 80]. Studies indicate HRQoL assessments are a broader measure of the impact of MS than tools measuring MS activity, and can identify elements of disease not considered by standard clinical tools [9, 79]. As the MSOC is a participant-centred intervention which aims to empower plwMS to self-manage their MS, HRQoL was considered a suitable primary outcome as it enables comprehensive measures of the effect of the disease, including aspects of health that cannot be evaluated using observer-based or clinical outcome measures. Importantly, HRQoL is most likely to show material and significant differences in the allotted timeframe of the study. Participants from the MSOC-feasibility trial who participated in two focus groups emphasised the importance of examining wellbeing and HRQoL in future studies (in addition to monitoring MS symptoms), further justifying HRQoL as a primary outcome [38]. However, changes in other clinical outcomes, such as disability and relapse rates, which are expected to take longer due to the time taken to affect relevant biological and inflammatory pathways [41], will also be examined. Notably, while acknowledging both HRQoL and other health outcomes may be influenced by factors not related to lifestyle changes, we anticipate the randomisation process will ensure factors not related to lifestyle will be equally represented between the IC and SCC groups.

## Conclusion

PlwMS have expressed the need for more information on lifestyle modification [81], and an online course could assist in providing consistent, comprehensive and consolidated information and in overcoming resource and accessibility barriers to provide this information. This RCT protocol ultimately aims to examine whether the MSOC is effective at improving the HRQoL and other health outcomes in plwMS. If found to be effective at one or more of these outcomes, the online IC will be made freely available via an MS charitable organisation which could be of immense benefit to members of the MS community. In the event there is minimal or no improvement in health outcomes, the quantitative and qualitative analyses performed as part of this study may inform future MS-related online course development.

## Data Availability

Not applicable.

## Abbreviations

DHQ: Dietary Habits Questionnaire
FSS: Fatigue Severity Scale
HADS: Hospital Anxiety and Depression Scale
HRQoL: Health Related Quality of life
IPAQ-SF: International Physical Activity Questionnaire - Short Form
MAQ: Mindfulness Adherence Questionnaire
MHC: Mental Health Composite
MS: Multiple Sclerosis
MSOC: Multiple Sclerosis Online Course
MSPSS: Multidimensional Scale of Perceived Social Support
PDDS: Patient-Determined Disease Steps
PHC: Physical Health Composite
PlwMS: People living with multiple sclerosis
QoL: Quality of life
RCT: randomised controlled trial
UWSE: University of Washington Self-Efficacy
